# Incisional keloid

**DOI:** 10.11604/pamj.2018.29.88.14375

**Published:** 2018-01-30

**Authors:** Pirabu Sakthivel, Chirom Amit Singh

**Affiliations:** 1 Department of Otorhinolaryngology & Head and Neck surgery, All India Institute of Medical Sciences, New Delhi, India

**Keywords:** Keloid, tympanoplasty, glucocorticoids

## Image in medicine

A 24-year-old man underwent left ear tympanoplasty for chronic otitis media and 3 months later started developing asymptomatic, tumor-like, cutaneous lesion over the incision site. There was no pain or itching. The lesion gradually progressed in the subsequent years protruding behind the ear to cause apparent cosmetic deformity. A clinical diagnosis of incisonal keloid was made. Keloids typically occur in response to dermal injuries such as surgical wounds, lacerations, burns, or inflammatory skin conditions. Because recurrence is common, surgical removal is generally not recommended; treatments with glucocorticoid injections, laser therapy, or radiotherapy may be tried but are usually associated with a poor response. Given the large size of the lesion in this patient, a complete surgical excision was performed and a diagnosis of keloid was confirmed histopathologically. The patient also received eight weeks infiltration of glucocorticoids postoperatively. There was no recurrence six months after surgery and the patient was lost to further follow-up.

**Figure 1:**
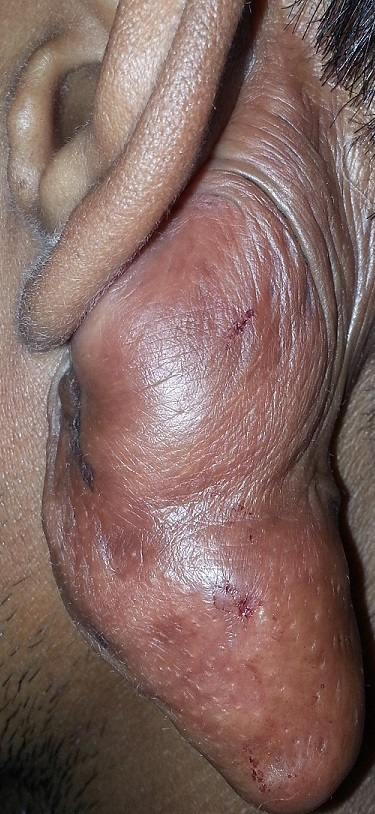
Clinical image showing classical postaural “incisional keloid”

